# Concerning global rise in measles cases

**DOI:** 10.1016/j.eclinm.2024.102502

**Published:** 2024-02-21

**Authors:** eClinicalMedicine

The UK Health and Security Agency declared a national incident on January 19, 2024, due to the rising number of measles cases in the country, with further warnings that the disease could spread. Despite achieving measles elimination status in 2021, there have been 216 confirmed cases and 103 probable cases in the West Midlands, UK, since October 2023, with most cases in children younger than 10 years. The USA, which had confirmed measles elimination in 2000, also reported 23 confirmed cases since December 2023, and over 30,000 cases were reported in the WHO European region between January and October, 2023. Moreover, provisional data indicate that, as of early December 2023, Yemen (25,216 cases), India (14,927 cases), Kazakhstan (12,985 cases), and Ethiopia (11,227 cases) are also experiencing outbreaks.

Measles is caused by a highly contagious and infectious airborne virus that can lead to severe disease and death, with unvaccinated young children, pregnant people, and those who are immunocompromised most at risk of complications. Before the introduction of a vaccine, measles was endemic throughout the world, responsible for 30 million cases and 2.6 million deaths each year. The first measles vaccine, based on the Edmonston strain-B, was licensed for public use in 1963, around which time individual countries introduced national vaccination campaigns. As a result of international efforts to control measles in Africa since 1966, The Gambia became the first country in the world to interrupt transmission of the virus. In the late 1960s, a more attenuated version of the vaccine, known as the Edmonston–Enders strain, was developed, which caused fewer severe side-effects than the first vaccine. This weaker strain was subsequently used to develop the combined measles, mumps, rubella vaccine (MMR), and a later version that also includes a varicella vaccine.

Community-wide vaccination is the most effective strategy for the prevention of measles, with an estimated 57 million deaths averted by measles vaccination worldwide during 2000–22. WHO recommends two doses of measles-containing vaccine for all children. At a population level, WHO advises at least 95% coverage of both doses to reach herd immunity. However, recent data published by WHO and the US Centers for Disease Control and Prevention suggest that global coverage is lagging, with only 83% of children having received the first dose of a measles-containing vaccine in 2022. This equates to an estimated 21.9 million children who did not receive their first dose (over half of whom were from ten countries [Nigeria, Democratic Republic of the Congo, Ethiopia, India, Pakistan, Angola, Philippines, Indonesia, Brazil, and Madagascar]), and 11 million children who did not receive their second dose. Despite a slight increase in global vaccination coverage from 81% to 83% during 2021–22, the number of reported global measles cases increased by 18%, deaths increased by 43%, and the number of countries experiencing large or disruptive outbreaks increased from 22 to 37. Most (63%) deaths during this period occurred in the WHO African region, followed by the Eastern Mediterranean region (29%), and the South-East Asia region (7%).

Progress towards the global elimination of measles was hindered by the COVID-19 pandemic, which led to suspensions or delays in disease surveillance strategies and vaccination campaigns worldwide, contributing to the latest outbreaks. Despite initial declines in measles cases as a result of social distancing measures, an estimated 61 million doses of measles-containing vaccine were missed or delayed during 2020–22 due to the pandemic, with global coverage of the first dose of measles-containing vaccine falling from 86% in 2019 to 81% in 2021—the lowest rate since 2008. Although global measles vaccination coverage data for 2022 suggest some recovery since the pandemic, coverage in low-income countries continues to decline.

Before the pandemic, vaccine hesitancy caused by misinformation on safety propagated by anti-vaccine groups, has also contributed to global measles outbreaks and deaths. Research suggests that over a third of individuals surveyed about childhood vaccination in Kazakhstan are vaccine hesitant, whereas declining rates of MMR vaccine coverage in Samoa before the 2019 outbreak were attributed to concerns over vaccine safety. Conflicts and wars also lead to an increase in refugees and displaced people, who are often excluded from national immunisation strategies, making them particularly vulnerable to infections.

To tackle the increasing global trend in measles cases, the Measles and Rubella Partnership published a strategic framework for 2021–30 to guide regional and national strategies and operational plans for achieving and sustaining measles and rubella elimination goals. These plans include addressing immunity gaps using approaches that are tailored to local challenges, embedding measles and rubella activities within immunisation and other primary health-care programmes, and improving surveillance efforts to enable more effective disease elimination and control activities. This year, the Gavi vaccine alliance also committed to supporting 15 low-income countries to implement measles and rubella vaccination campaigns.

We are witnessing an alarming rise in global measles cases and deaths. Low-income countries carry the largest burden, reflecting the declining vaccination coverage rates. By strengthening global surveillance strategies, renewing efforts to ensure that all children are fully vaccinated, and developing targeted campaigns to promote the safety and efficacy of measles vaccines, as well as encourage any individuals to come forward to receive their missed vaccine doses, we can overcome the setbacks caused by the COVID-19 pandemic, and prevent further outbreaks and transmission of the disease.

